# Proteomic research of proteins involved in pain expression in an animal model of chronic pain

**DOI:** 10.1186/1129-2377-16-S1-A8

**Published:** 2015-09-28

**Authors:** Elisa Bellei, Stefania Bergamini, Emanuela Monari, Aurora Cuoghi, Michele Zoli, Aldo Tomasi, Maria Michela Cainazzo, Simona Guerzoni, Luigi Alberto Pini

**Affiliations:** Department of Diagnostic Medicine, Clinic and Public Health, Proteomic Lab, University of Modena and Reggio Emilia, Modena, Italy; Department of Biomedical, Metabolic and Neural Sciences, University of Modena and Reggio Emilia, Modena, Italy; Inter Department Headache and Drug Abuse Center, University of Modena and Reggio Emilia, Modena, Italy

## Background

Pain is defined as an unpleasant sensory and emotional experience, associated with a real or potential tissue damage. Chronic pain (CP) can arise from tissue injuries or inflammation (inflammatory pain), or from lesions of the peripheral or central nervous system (neuropathic pain). The purpose of this study was to search proteins potentially involved in the expression or mediation of pain utilizing proteomic techniques, in an animal model of CP obtained by the ligation of the sciatic nerve.

## Methods

The rats (Wistar) used in this study were divided into 4 groups: two control groups, one treated with saline and the other with Indomethacin (2 mg/kg for 3 consecutive weeks), and two groups with the ligation of the sciatic nerve[1], subjected to the same treatment. Initially, two behavioral tests (“Plantar test” and “Von Frey test”) were carried out to confirm the presence of pain in operated animals. Subsequently, the proteomic analysis was performed on serum samples, first by one-dimensional protein separation (SDS-PAGE) and then by two-dimensional gel electrophoresis (2-DE). The differentially expressed proteins among the different groups were identified by mass spectrometry (ESI-Q-ToF/MS).

## Results

The most significant result obtained by SDS-PAGE analysis was observed in the group of operated rats treated with saline, where the expression of 6 protein bands was significantly increased, compared to rats treated with Indomethacin. The 2-DE analysis confirmed these data and allowed to identify 6 additional different proteins, which included typical inflammatory proteins, antioxidant enzymes and proteins with neuroprotective function, implicated in the degeneration/regeneration of peripheral nerves (e.g., ApoE). Particularly, a prostaglandin with a pivotal role in central sensitization, involved in induction of hyperalgesia and cutaneous allodynia, was identified. This protein was also detected in our previous study on medication-overuse headache (MOH) patients, where we found it significantly increased in urine of NSAIDs, mixture and triptans abusers, in respect to the healthy control group[2].

## Conclusions

The presence of these proteins may be due to an attempt of functional recovery of the nerve and, at the same time, of pain reduction. For this reason, they could be a potential target for the understanding and treatment of peripheral neuropathy. These findings need to be validated by other experiments, such as using molecular biology techniques.
